# Poster Session II – Poster of Distinction II - A213 IN UTERO EXPOSURE TO BIOLOGIC THERAPIES AND EARLY INFANT HEALTH OUTCOMES AMONG CHILDREN BORN TO MOTHERS WITH INFLAMMATORY BOWEL DISEASE: A PROSPECTIVE STUDY

**DOI:** 10.1093/jcag/gwaf042.213

**Published:** 2026-02-13

**Authors:** C Fung, V Srikanth, K O’Connor, V W Huang

**Affiliations:** University of Toronto Temerty Faculty of Medicine, Toronto, ON, Canada; Sinai Health, Toronto, ON, Canada; Sinai Health, Toronto, ON, Canada; University of Toronto, Toronto, ON, Canada

## Abstract

**Background:**

Biologic therapies represent an important therapeutic avenue for inflammatory bowel disease (IBD) during pregnancy, and current guidelines recommend continuation of biologics throughout pregnancy. There are only a few studies following infants exposed to biologics in utero.

**Aims:**

This study aimed to evaluate the relationship between in utero biologic exposure and early-life health outcomes in infants born to mothers with IBD.

**Methods:**

We conducted a prospective cohort study of 145 mother-infant pairs from a tertiary care center. Biologic exposure during pregnancy was defined as documented maternal use of biologic therapies at any point during gestation. Infant outcomes assessed included birthweight, low birthweight (<2500g), gestational age, preterm birth (<37 weeks), APGAR scores, NICU admission, incidence of infections, hospitalizations, and allergy/atopy outcomes during the first year of life. Analyses included t-tests for continuous outcomes and Chi-square/Fisher’s Exact tests for categorical outcomes.

**Results:**

Among 145 infants, 94 (65%) were exposed to biologics in utero. Mean birthweight did not significantly differ between biologic-exposed and unexposed infants (3282g vs. 3348g, *p*=0.501), nor did gestational age or 1- and 5-minute APGAR scores. Biologic-exposed infants had a higher rate of NICU admission compared to unexposed infants (17% vs. 4%, *p* = 0.007; Fisher’s Exact *p* = 0.006), though this association did not remain significant after correction for multiple comparisons. The proportion of infants classified as low birthweight trended higher in the biologic-exposed group (17% vs. 3%, *p*=0.043, Fisher’s *p*=0.050), though results fell below significance thresholds after correction. No significant differences were found in rates of infection, hospitalization, or allergy within the first year of life. Additional analyses assessing maternal disease complexity including history of fistulizing disease, surgery, or perianal involvement did not reveal any significant associations with infant health outcomes.

**Conclusions:**

While most early infant outcomes were similar, biologic exposure in utero was associated with increased rates of low birthweight and NICU admission. These findings warrant further prospective investigation. Clinicians should weigh the maternal benefits of biologic therapy against possible neonatal risks and ensure appropriate pediatric follow-up for exposed infants.

**Funding Agencies:**

CAG, CIHR

